# Reimagining Day Rehabilitation For Frailty and Neurodegenerative Conditions through the integrated Rehabilitation and EnAblement Program (iREAP)

**DOI:** 10.5334/ijic.8066

**Published:** 2024-09-17

**Authors:** Genevieve Maiden, Annabel Kingsford, Audrey P. Wang, Anh R. Tran-Nam, Julia Nelson

**Affiliations:** 1 Uniting War Memorial Hospital, Waverley, Australia; 2 Biomedical Informatics and Digital Health, School of Medical Sciences, University of Sydney, Australia; 3 DHI Lab, Research Education Network, Western Sydney Local Health District, Westmead Health Precinct, Westmead, NSW 2145, Australia; 4 Macquarie University Clinical School, Faculty of Medicine, Health and Human Sciences, Macquarie University, Sydney, NSW, Australia; 5 Northern Beaches Hospital, Sydney, NSW, Australia

**Keywords:** frailty, neurodegenerative disease, parkinsons disease, multidisciplinary, day rehabilitation

## Abstract

**Background:**

integrated Rehabilitation and EnAblement Program (iREAP) is an innovative redesign of the traditional day rehabilitation model, providing an anticipatory, early assessment and intervention program that manages care of community-dwelling older people with complex needs. It coordinates access to disciplines across medical, allied health and nursing, with a self-management focus, partnering with primary health in an integrated approach.

**Objective:**

This observational study reviews the effectiveness of iREAP on frailty, patient activation, quality of life and physical outcome measures on older people at risk of, or experiencing falls and frailty, or with neurodegenerative conditions, including Parkinson’s Disease.

**Methods:**

99 participants completed the eight-week multidisciplinary program. Patient outcome measures included Rockwood Clinical Frailty Scale, quality of life measures, Patient Activation Measure, Timed Up and Go, 6 Minute Walk Test and Berg Balance Scale.

**Results:**

On completion of iREAP, participants displayed improvements in their Rockwood Clinical Frailty Scores (mildly frail to vulnerable), ‘patient activation’ (55.08 to 60.61), quality of life (Parkinson’s Disease Questionnaire-39, 49.93 to 47.16; WHO Quality of Life – Bref physical domain, 21 to 22.7) and physical measures including balance (44 to 49/56 Berg Balance scale) and mobility (294 m to 336 m, 6-minute walk test). Falls were not reduced at twelve months post-program (3.40 to 2.01).

**Conclusion:**

iREAP is an interdisciplinary, early assessment and intervention program with the potential to reverse frailty and improve quality of life for complex older patients. This paper offers a platform for future research, given the paucity of evidence reviewing the efficacy of integrated anticipatory models of care in older adults with complex needs.

## Introduction

A central consideration in the healthcare of older people is the complexity of their care needs [1]. Many older people live with multiple chronic illnesses, including Parkinson’s Disease (PD) and other geriatric syndromes, affecting patients across medical, functional, and psychosocial domains [1]. Complex health issues see older people vulnerable to falls and frailty, which themselves are common major geriatric health problems [2,3]. This often leads to older people feeling disempowered in managing their own health [4].

Unfortunately, the majority of community-based healthcare services for older people in Australia are provided by a range of organisations with variable linkages and are predominantly accessed in a reactive manner [4]. This leads to multiple and sometimes redundant health system interactions, alongside delays and missed opportunities to access appropriate care [4]. There is a recent emphasis towards integrated care for older people (ICOPE), as advocated by the World Health Organisation [5]. At a local, state and national level, ICOPE is highlighted as an approach to better meet the complex needs of older people [4,6].

Day hospitals or day interdisciplinary rehabilitation programs have been proposed as a method of meeting these complex needs [7]. However, despite the intuitive benefits of day rehabilitation, a 2015 systematic review of 16 randomised and quasi-randomised trials comparing day hospital care for older patients versus other forms (comprehensive care, home care or no comprehensive care), concluded there is only low-quality evidence that day hospital care is superior to non-comprehensive geriatric care [8]. This may be because these studies reflect the traditional model of care for day rehabilitation programs, which are typically considered a complement to inpatient care, with a focus on physical outcomes as opposed to an interdisciplinary integrated approach [9].

**Figure 1:**
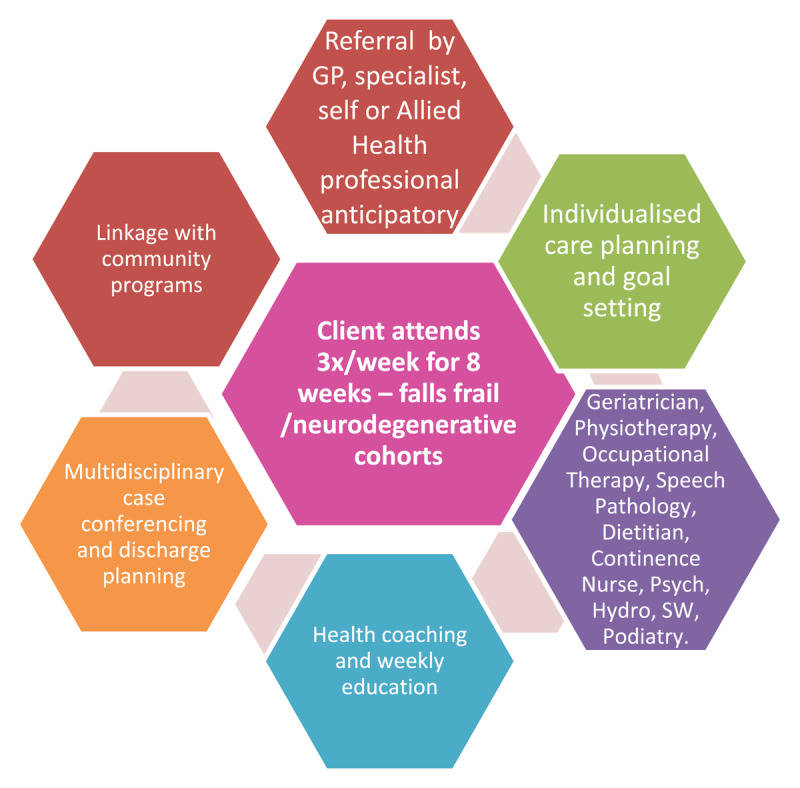
iREAP Model of Care.

In contrast, the integrated Rehabilitation and EnAblement Program (iREAP), delivered at Uniting War Memorial Hospital (UWMH), reimagines the traditional day rehabilitation model, providing anticipatory, coordinated and interdisciplinary intervention (See Figure 1). iREAP provides a flexible and integrated model of care addressing the individual’s holistic needs. The program consists of an eight-week intervention for community-dwelling older people who are becoming increasingly frail, experiencing falls, or with a neurodegenerative disorder such as PD. iREAP translates evidence-based principles of individualised care planning, patient-generated goal setting, and anticipatory health coaching into clinical practice. The model implements early intervention for both frailty and neurodegenerative conditions [10,11]. It also provides ‘value-based care,’ delivered in an integrated and person-centred way, emphasising the individual at the centre of decision making and care planning [6] (Table 1).

**Table 1:** Reimagining Day Rehabilitation – evolution of the iREAP model of care.


TRADITIONAL DAY HOSPITAL	iREAP

Post-acute referral pathways	Referral direct from primary care, specialist physicians, Aged Service Emergency Team (ASET), community allied health and self-referral

Post crisis, post illness, post-injury, post-surgical intervention	Anticipatory – pre-crisis intervention for those at risk of, or experiencing frailty and/or falls; or early in course of a neurodegenerative condition

Focus on physical outcomes and ADL’s	Holistic across physical, psychological and social domains

Fixed treatment protocol	Individualised care plan related to the individual’s goals of care

Didactic education approach	Health coaching, education and enablement

Single discipline lead	Geriatrician and Care Coordinator lead Multi-disciplinary case conferencing and intervention

Discharge home exercise program	Individualised enablement strategies through health coaching process

Physical outcome measures	Outcome measures across all health domains


## iREAP Model of Care

Over an eight-week period, appropriate participants attend iREAP for three half days per week and are assessed individually with ongoing treatment as required by the multidisciplinary team including geriatrician, physiotherapy, occupational therapy, dietetics, podiatry, social work, speech pathology, hydrotherapy, continence nursing and psychology. Participants attend a series of group exercise classes and education sessions (specific to each group), and occupational therapy functional groups. The aim of education is to build health literacy, knowledge and confidence with self-management and includes the additional disciplines of pharmacy and Parkinson’s Disease counsellor. Individualised goal setting and health coaching is a key element. On discharge, participants are referred to appropriate community services, classes or programs, alongside individualised exercise and management plans.

The iREAP care coordinator provides a key role within the iREAP program. Beyond the screening and assessment process, the coordinator interacts with participants during and between visits to provide extra assistance, including care coordination, carer support and practical advice (e.g. organising transport). Follow-up contact is also made post program by the care coordinator to discuss participants engagement with enablement strategies and recommendations. All health professionals use a health coaching approach within the program, and the care coordinator runs specific health coaching sessions in a group setting to enable peer discussion on ways to maintain health engagement strategies beyond the program. If present, informal carers are invited to participate in this, along with the education sessions; however in practice, most choose to use the time for respite purposes.

UWMH is a subacute aged rehabilitation facility, with inpatient, outpatient and community programs, offering pathways from primary health and acute care for those in functional decline and with escalating dependence [12]. iREAP was developed to address a gap in pre-crisis coordinated intervention, for those whose function is deteriorating and impacting quality of life. Unlike home-based rehabilitation and rapid response care [12], iREAP offers potential economies of scale in providing interdisciplinary input through its base in outpatient services in a group format. It also leverages the benefits of social and peer support, utilising health coaching principles to guide the group to their own solutions. Participants describe the building of networks and support relationships which continue beyond the program.

## Methods

### Aim

The aim of the study was to assess the effectiveness of iREAP in improving physical outcomes, quality of life and self-management in older people with complex needs, and by doing this, provide a viable alternative for delivering an anticipatory, interdisciplinary and holistic model of care.

### Study design

This observational study was conducted for the purposes of quality improvement and service evaluation and was thus a pre-post uncontrolled study design.

The target population included community-dwelling, older people with frailty with complex health problems who were at risk of falls, hospitalisations and residential care placement or who had a neurodegenerative disease. Participants were allocated by primary diagnosis into either a Falls Frail (FF) or Neurodegenerative, (predominantly Parkinson’s) (ND) group to facilitate administration of specialised Parkinson’s and neurodegenerative therapies (e.g. LSVT BIG® and LSVT Loud®) [13,14].

Inclusion criteria was specific to the participants’ condition. For the FF group, participants were accepted if they had fallen in the last twelve months, were at risk of falls as determined by the referrer, experienced multiple hospitalisations, were pre-frail or frail. For the ND group, participants had a diagnosis of mild to moderate neurodegenerative disease e.g. Parkinson’s or a Parkinson’s plus syndrome. All participants were independent with toileting and mobility (with or without aid) or had a carer to assist. Participants were primarily referred through primary care (via General Practitioners), as well as local hospitals, emergency departments, specialists, and self-referrals. The care coordinator screened referrals via phone and an initial physiotherapy assessment functioned as a secondary screening point.

Age, sex, cognitive status, and presence of polypharmacy were collated through electronic medical records review, extracted from the comprehensive geriatric and care coordinator assessments. Polypharmacy, commonly defined as five or more medications, was the definition adopted in this study [15]. Dementia was defined as a diagnosis by geriatrician or other specialist, and Mild Cognitive Impairment (MCI) as a Montreal Cognitive Assessment (MoCA) <25 without a diagnosis of dementia or functional impairment. Dementia was not an exclusion criterion if otherwise able to engage, with a carer present if needed. Physical measures were completed at the initial physiotherapy assessment and other outcomes measured during week one, with all measures repeated in the final week.

Frailty was assessed using the Rockwood Clinical Frailty Scale (CFS) [16] and falls risk using physical measures of Timed Up and Go (TUG) [17] (15 seconds considered the cut off for older adults already attending a falls clinic) and Berg Balance Scale (A Berg Balance Scale of 45 and above, shown to reduce a participant’s risk of falling) [18]. Mobility endurance was measured using Six Minute Walk Test (6MWT) [19].

The Patient Activation Measure (PAM-13) is a patient-reported outcome assessing the knowledge, skill and confidence in the management of one’s health [20], giving a measure of ‘patient activation’. Quality of Life was measured using the World Health Organisation Quality of Life tool (WHOQOL- Bref), and the Parkinson’s Disease Questionnaire (PDQ – 39) as an additional specific measure for PD.

Self-reported falls within the twelve months prior were recorded using the question ‘In the last twelve months have you had any falls?’ Follow-up via phone at twelve months post program recorded self-reported falls, questioning ‘Since attending the program have you had any falls?’ Twelve month follow up was considered the optimal follow up time to enable greater accuracy and recall of falls [21,22].

The de-identified data was analysed by an independent researcher who was not involved in the data collection or iREAP service provision.

### Statistical analysis

All data was checked for normal distribution including skewness and kurtosis with α <0.05. Self-reported falls data was not normally distributed, so a non-parametric test was chosen. The Wilcoxon signed rank test was used in SPSS version 23, with the null hypothesis that the median difference between pre and post data equalled zero. A p-value of <0.05 was used to test significance. CFS data was analysed using marginal homogeneity test and effect sizes reported. All other data was analysed with paired t-tests. Multiple comparisons were controlled for, using the Benjamini-Hochberg procedure set at a 2% false discovery rate and each resulting p-value compared against a critical p-value [23].

### Ethics

Ethics review conducted by South East Sydney Local Health District Human Research Ethics Committee approved the study as quality improvement or quality assurance activity not requiring independent ethics review.

## Results

### Participants

A total of 108 consenting participants commenced iREAP during the study period. 99 (91.6%) participants completed the program in full. 52 participants were allocated to the ND group, and 47 to the FF group. Non-completers of the program (n = 9) dropped out due to unrelated acute medical illness. Of the 99 program completers, a complete data set of pre and post measures was completed on 68 (69%) participants. Reasons for incomplete measures included not attending when outcome measures were collected, and not completing or returning questionnaires in full.

The average age of participants was 76 years, FF group mean age was 80 years old compared with the ND group at 72 (See Table 2). There was a balanced sex ratio for the FF group, however male predominance (67.3%) in the ND group was consistent with the higher rates of males diagnosed with ND, usual ratio of 2:1 [24]. Overall, 25 (25%) patients had a diagnosis of dementia, with a further 26 (26.3%) having MCI. There was an increased rate of dementia in the ND group (30.8%) compared with the FF group (19.1%).

**Table 2:** Baseline Characteristics of Participants.


CHARACTERISTIC	TOTAL GROUP (n = 99)	FALLS/FRAIL GROUP (n = 47)	ND GROUP (n = 52)

Age (years)			

Mean	76	80	72

Range	57–91	57–91	59–85

Sex, n (%)			

Female	41 (41.4%)	23 (48.9%)	17 (32.7%)

Male	58 (58.6%)	24 (51.1%)	35 (67.3%)

Cognition, n (%)			

Intact	38 (38.4%)	24 (51.1%)	14 (26.9%)

MCI	26 (26.3%)	11 (23.4%)	15 (28.8%)

Dementia	25 (25.2%)	09 (19.1%)	16 (30.8%)

Unknown	010 (10.1%)	03 (06.4%)	07 (13.5%)

Polypharmacy, n (%)			

Present	60 (60.6%)	17 (36.2%)	35 (67.3%)

Not present	31 (31.3%)	25 (53.2%)	14 (26.9%)

Unknown	08 (08.1%)	05 (10.6%)	03 (5.8%)


**Figure 2:**
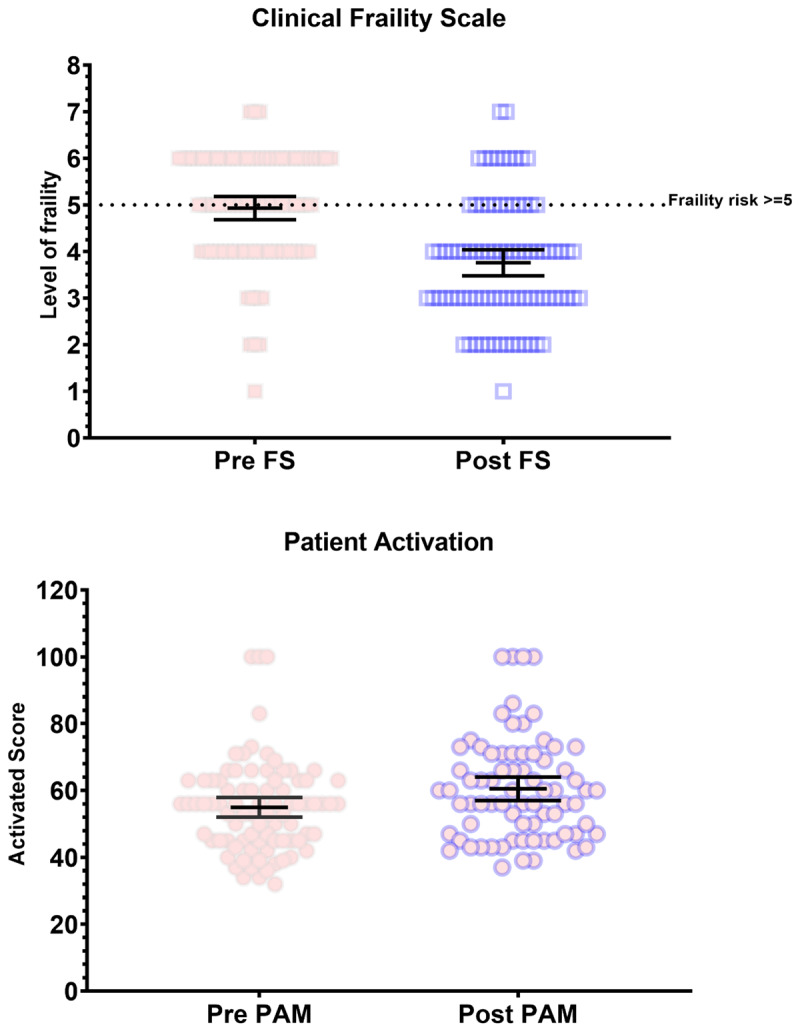
Clinical Frailty Scale and Patient Activation Measure (PAM-13). *Pre-FS – pre-program CFS; Post FS – post-program CFS.

The CFS scores (n = 84) showed a statistically significant improvement from pre-program median score of 5 (mildly frail) to a post-program median score of 4 (vulnerable) (p = 0.0001) (Figure 2). This statistically significant improvement in frailty is deemed clinically relevant as a score ≥5 is considered frail [16,25,26]. This reflected a change for 35 clients, with a shift from a category of 5 or more pre-iREAP to less than 5 post-iREAP, thus no longer considered frail.

The mean patient activation score as measured by the PAM-13 showed statistically and clinically significant improvement pre-iREAP from 55.08 to 60.61 post-iREAP (n = 80) (Figure 2). These values move participants from Level 2 (PAM score 47.1–55.1), where the participant “lacks knowledge and confidence to take action” in terms of activation and health empowerment, to a Level 3 (55.2–67.0), “beginning to take action” which includes “striving for best-practice behaviours” and “feeling part of their healthcare team” [27,28]. In practice, this suggests patient have more control and ownership over their health and health outcomes and are beginning to take action to implement these behaviours.

### 6-minute walk test (6MWT)

6MWT improved significantly from 294 m pre-iREAP to 336 m post-iREAP (n = 69; p = 0.001) (Table 3). This improvement is clinically significant in this geriatric population, with 20m considered to be a small meaningful change, while a substantial meaningful change is considered to have occurred with an improvement of 50m [26].

**Table 3:** Physical measures.


PHYSICAL TEST		MEAN	RANGE (min–max)	*P-VALUE*

Timed Up and Go in 3m (*s*)	Pre	16.99	4.79–62.00	0.007

	Post	14.25	4.97–49.52	

Berg Balance*	Pre	44.44	15–56	0.00002

	Post	48.54	21–56	

6-minute walk test (*m*)	Pre	293.81	43.5–605	0.001

	Post	336.06	133–600	


* The Berg Balance Scale total maximum score of 56 indicates no balance issues detected.

### Berg Balance Scale

Berg Balance Scale (n = 90) showed statistically significant improvement from a mean pre-iREAP score of 44/56 to a mean post-program of 49/56, indicating a reduced falls risk (Table 3) A Berg Balance Scale of 45 and above has shown to reduce a participant’s risk of falling [18,29]. This was reflected in a change for 11 clients balance score moving from below 45 to above 45.

### Time Up and Go (TUG)

TUG showed statistical significance, improving from pre-program mean time of 16.99 seconds to post-program mean time 14.2 seconds (n = 33; p = 0007) (Table 3).

### Quality of Life Questionnaires

The WHOQOL-Bref (n = 39) showed a statistically significant improvement in the physical domain post-program (22.7) compared to pre-program (21) (p = 0.005). While there was improvement in the other domains, these did not reach statistical significance. PDQ-39 index (n = 73), targeted specifically at the ND group, improved significantly from pre-program 49.93 to post-program 47.16 (p = 0.004) (where a lower score indicates an improved QOL).

### Subjective report of Falls

A drop-out rate at phone follow-up of less than ten percent at 12 months was achieved, with post data available for 92 participants. The mean number of falls, although reduced at 12 month follow up (3.40 pre, to 2.01 falls post-program), did not show statistical significance.

## Discussion

Despite the limitations of the observational design, the analysis provides encouraging results on the effect of iREAP on frailty, quality of life, physical measures and patient activation. Our study identified that participants are less frail after participating in iREAP, with the median CFS score decreasing from ‘mildly frail’ to ‘vulnerable’. The baseline mean CFS score was relatively low (“mildly frail”), which is commensurate with the design of the program as an anticipatory intervention targeting at-risk older people still living in the community. A single category change in CFS among community- dwelling older people increases the risk of death and residential care placement by 21.2% and 23.9% over an approximate 6-year period [16,25]. The improvement in CFS supports the body of evidence that targeted intervention can enable reversibility of frailty [25,26]. Future study could determine if this improvement is maintained twelve months post-program.

The study identified statistically significant improvements in the TUG and 6MWT, demonstrating improved mobility and endurance. BBS demonstrated statistically and clinically significant improvement, as participants’ risk of falling is reduced with a score of 45 or above. While there was a statistically significant improvement in mean TUG, it did not reduce to less than 14 seconds, the cut-off often considered consistent with reduced falls risk [29,30,31,32]. However, cut-off values evidenced in other literature include 15 seconds for older adults already attending a falls clinic [31], 32.6 seconds in the older person with frailty [30] and 7.95–11.5 seconds in ND [34,35,36]. Findings from systematic reviews and meta- analyses also suggest TUG has use in discriminating fallers from non-fallers in less-healthy, lower-functioning groups [33]. Thus, the findings could be considered clinically significant.

WHOQOL-Bref has four domains, one being the physical domain, which showed statistically significant improvement which increased from 21.00 to 22.67 (p = 0.006). This may speak to participants’ focus on goals related to physical activity through group and individual exercise sessions. The PDQ-39 assesses PD-specific symptoms impacting on a person’s function and despite PD being degenerative in nature, participants showed a statistically significant improvement in QOL from 49.93 to 47.16 (p = 0.004). In terms of understanding longer term impacts across domains in PD, repeat examination at 12 months would be recommended.

Post iREAP, participants had improved ‘activation’ in terms of their healthcare as measured by PAM-13, which demonstrated statistically and clinically significant improvement from 55.08 to 60.61. This change takes participants into a higher category in terms of their ‘knowledge, skills and confidence’ in managing their health. A study of multimorbid older people found increases in PAM-13 score are correlated with higher levels of best practice health behaviours [20]. This implies iREAP participants are more able to independently manage changes in their health into the future and feel part of their health care team. An improvement of five points in PAM-13 leads to approximately 10.5% and 6.5% respective increases in levels of physical activity and medication adherence [20]. It is hypothesised that the strong emphasis on health coaching as part of iREAP contributed to these improvements.

Although the BBS score showed clinical improvements for balance overall, there was no similar finding for the subjective reporting of falls at 12 months compared to 12 months prior. However, in sub-group exploratory analysis the FF group demonstrated a statistically significant reduction in the number of falls pre and post program. The neurodegenerative (ND) group also showed a reduced number of falls, however this did not reach statistical significance, an anticipated finding as falls increase over time a result of degenerative disorders [36]. Notably, self-reporting of falls is often subject to recall bias by the subject [37] and given the percentage of iREAP clients with cognitive impairment, this is a pertinent consideration. Polypharmacy was present in 60.6% of participants, and higher in the ND group (67.3%). Evidence demonstrates that polypharmacy is a predictor of falls, and that Geriatric Assessment including reconciliation of medication, alongside pharmacy education and ongoing management through the GP and community pharmacist, is an essential element of care. This was therefore an integral aspect of iREAP.

Future research could include a randomised controlled trial that would introduce greater experimental rigor to assess effectiveness of iREAP compared to a control group in sustaining outcomes at a year post program. This paper supports policy and practice which focuses on anticipatory multidisciplinary care options which keep people well in the community and out of hospital. It demonstrates a model which can be implemented in an outpatient setting, integrated through strong links with primary health.

In practice, the broad iREAP inclusion criteria are favourable, demonstrating change in a representative sample of an older community-dwelling cohort. A systematic review showed rates of dementia in PD to be as high as 24–31% [38] while dementia in the general population in Australia is 10% for those over 65, increasing to 30% for those over 85 years [39]. This study did not exclude those with dementia or cognitive impairment, a novel demonstration that with appropriate supports, this cohort with cognitive impairment can participate in, and benefit from, an interdisciplinary program.

## Limitations

As an observational study conducted primarily for the purposes of qualitative improvement, results should be interpreted with caution. It is a single centre design, and assessors were not blinded during data collection and were at times involved in the clinical care of participants. Inter-rater variability could be present as assessors were not consistent. There was an attempt to improve the integrity and completion of data collection through a dedicated research assistant for data collection and blinding for statistical data analysis.

## Conclusion

As Australia’s ageing population grows, our health system must deliver early intervention models to improve outcomes for older people, enabling direct pathways from primary health. As the system focuses on acute beds and managing crises, we often neglect the opportunity to proactively manage and intervene in the subacute space when participants are declining and moving into a more dependent phase of care. iREAP shifts the focus from reactive healthcare, to one which anticipates peoples’ needs with the aim of arming them with knowledge, skills and confidence to manage longer term. Scale and spread of iREAP is achievable in an ambulatory care setting, via a clinical redesign approach, with a care coordinator key to enabling integration of the interdisciplinary team including primary health and community enablement programs. It is important that the participant is a partner in their care, with a goal-orientated and holistic approach the target.

Overall, iREAP presents a novel model of care for a different day rehabilitation approach. It is anticipatory and comprehensive, and this study suggests it reduces frailty and improves patient activation, quality of life, physical ability and balance. iREAP provides a viable alternative, enabling greater numbers of older people to continue living safely in the community, improving the patient journey and experience through pre-crisis intervention, and potentially reducing acute health care costs and hospitalisations.
